# Phytotoxicity and hormesis in common mobile organic compounds in leachates of wood-derived biochars

**DOI:** 10.1007/s42773-024-00339-w

**Published:** 2024-05-22

**Authors:** Sean C. Thomas, Ryan Ruan, Nigel V. Gale, Sossina Gezahegn

**Affiliations:** Institute of Forestry and Conservation, John H Daniels Faculty of Architecture Landscape and Design, University of Toronto, 33 Willcocks St., Toronto, ON M5S 3B3 Canada

**Keywords:** Acetic acid, Caproic acid, Charcoal, Carboxylic acids, Germination, Pyrogenic carbon, Pyroligneous acid, Valeric acid, Volatile fatty acids, Wood vinegar

## Abstract

Although addition of pyrolyzed organic materials (biochars) to soil generally results in increased growth and physiological performance of plants, neutral and negative responses have also commonly been detected. Toxicity of organic compounds generated during pyrolysis, sorbed by biochars, and then released into the soil solution, has been implicated as a possible mechanism for such negative effects. Conversely, water-soluble biochar constituents have also been suggested to have “hormetic” effects (positive effects on plants at low concentrations); however, no specific compounds responsible have been identified. We investigated the relative phytotoxicity—and possible hormetic effects—of 14 organic compounds common in aqueous extracts of freshly produced lignocellulosic biochars, using seed germination bioassays. Of the compounds examined, volatile fatty acids (VFAs: acetic, propionic, butyric, valeric, caproic, and 2-ethylbutyric acids) and phenol, showed acute phytotoxicity, with germination-based ED50 values of 1–30 mmol L^−1^, and 2-ethylbutyric acid showed ED50 values of 0.1–1.0 mmol L^−1^. Other compounds (benzene, benzoic acid, butanone, methyl salicylate, toluene, and 2,4-di-tert-butylphenol) showed toxic effects only at high concentrations close to solubility limits. Although phytotoxic at high concentrations, valeric and caproic acid also showed detectable hormetic effects on seedlings, increasing radicle extension by 5–15% at concentrations of ~ 0.01–0.1 mmol L^−1^. These data support the hypothesis that VFAs are the main agents responsible for phytotoxic effects of lignocellulosic biochar leachates, but that certain VFAs also have hormetic effects at low concentrations and may contribute to positive effects of biochar leachates on early plant development in some cases.

**Graphical Abstract:**

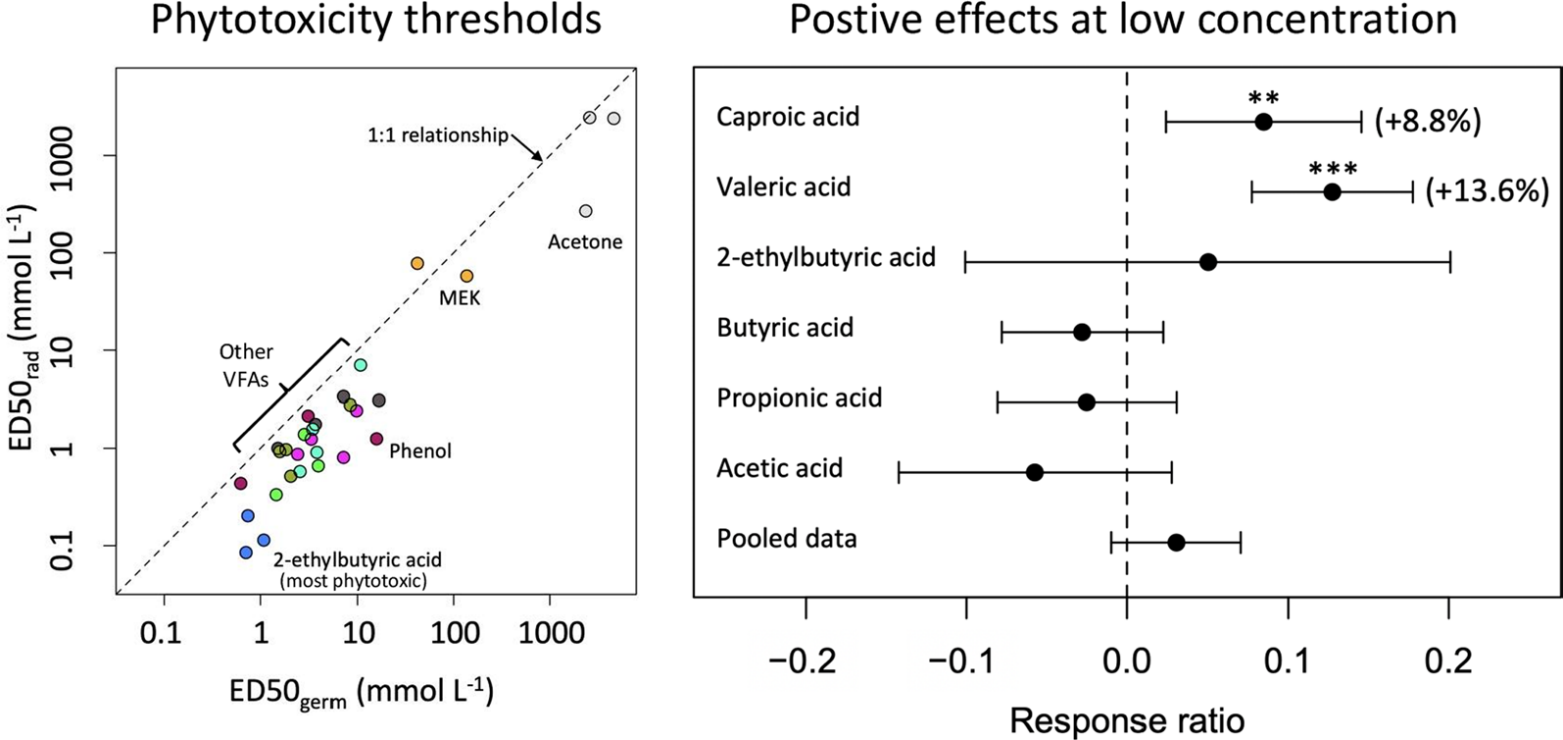

**Supplementary Information:**

The online version contains supplementary material available at 10.1007/s42773-024-00339-w.

## Highlights

- 151 compounds were identified in leachates from 13 biochars.
- Among common compounds, volatile fatty acids (VFAs) and phenol showed the most pronounced phytotoxic effects.
- Some VFAs also had hormetic effects, enhancing radicle extension growth at low concentrations.
- Effects were consistent among test plant species but the smallest-seeded species showed the highest sensitivity.

**Supplementary Information:**

The online version contains supplementary material available at 10.1007/s42773-024-00339-w.

## Introduction

Although scientific study of the use of pyrogenic carbon or charcoal as a soil amendment extends back to the 1800s (Wilson 2014; Thomas and Gale 2015), modern work on the subject commenced only recently, coincident with the coining of the term “biochar” as pyrolyzed organic matter intended specifically for this use (Lehmann et al. 2006). The promise of biochar as a beneficial soil amendment has been supported by recent meta-analyses: growth and yield responses in agronomic systems generally average ~ 10–30% (Biederman and Harpole 2013; Liu et al. 2013; Jeffery et al. 2017; Dai et al. 2020; Ye et al. 2020; Joseph et al. 2021), and an average response of 41% in growth has been found in trees (Thomas and Gale 2015). However, these average responses belie considerable variability. Biochars vary greatly in chemical and physical properties depending on feedstock and pyrolysis conditions (Kloss et al. 2012; Chia et al. 2015; Gezahegn et al. 2019), and this variability, along with differences due to dosage (Gale and Thomas 2019), contributes to high variation in plant responses among biochars (e.g., Rajkovich, et al. 2012). There is likewise considerable variability in responses among different plant species treated with the same biochar (e.g., Pluchon et al. 2014; Gale et al. 2017).

Implementing biochar operationally demands a detailed understanding of the mechanisms responsible for responses of plants (Joseph et al. 2021). In this regard an important generalization is not only that plant growth responses to biochar are variable, but also that negative responses are common. Spokas et al. (2012), in a “vote-counting” review, concluded that roughly 20% of biochar trials resulted in negative plant growth responses. This value may overstate the occurrence of negative effects, since meta-analyses make clear that there is a predominant positive trend albeit with high variation (e.g., Liu et al. 2013; Thomas and Gale 2015). Nevertheless, there are well-documented cases of negative growth responses to biochars in individual studies (e.g., Rajkovich et al. 2012; Gale et al. 2016, 2017; Sarauer and Coleman 2018).

Several potential mechanisms for such negative effects have been hypothesized. Biochar feedstocks can contain potentially toxic elements that are then concentrated in biochars produced. For example, biochars made using food waste as a feedstock can have pronounced negative effects on plant growth due to high sodium (Rajkovich et al. 2012). Likewise, high-carbon wood ash biochars can be high in metals such as Pb, Cd, and Cu (e.g., Bieser and Thomas 2019). Alternatively, the liming properties of biochars can act to limit plant nutrient availability, particularly on soils that are already alkaline (e.g., Sarauer and Coleman 2018). In addition, biochars are generally low in N, and what N is present is often covalently bound or otherwise not readily available (Clough et al. 2013). Moreover, biochars generally strongly sorb ammonium ions, and can thus reduce mineralized N availability (Clough et al. 2013; Wang et al. 2015), resulting in negative growth responses in plant species sensitive to N limitation (Gale et al. 2017).

Another mechanism that may commonly account for cases of negative effects of biochars on plant performance is the presence of organic compounds that are produced during pyrolysis, and then sorbed by biochars (Spokas et al. 2010, 2011). Most of these organic compounds are “mobile”, being unbound to the graphitic skeleton of biochar and either water-soluble and/or volatile (Buss and Mašek 2014; Buss et al. 2015). Toxic effects of aqueous extracts of biochar found for a variety of biochars have been attributed to such mobile organic compounds. In addition to inhibitory effects on plant germination (Rogovska et al. 2012; Buss and Mašek 2014; Kołtowski and Oleszczuk 2015), and later growth (Gale et al. 2016), mobile organic compounds have been implicated in toxic effects of biochar leachates on soil microbes (Lehmann et al. 2011), and on other organisms including protozoa and freshwater invertebrates (e.g., Oleszczuk et al. 2013; Flesch et al. 2019). Phytotoxic effects vary appreciably among plant species, suggesting that relative sensitivity to phytotoxic compounds could contribute to high interspecific variation in biochar effects on early plant growth (Gale et al. 2016).

A wide variety of mobile organic compounds have been detected in biochars. Spokas et al. (2011) assessed 80 biochars and identified a total of 160 organic compounds, though with only 8 compounds common to at least half of the biochars tested. There are additional organic compounds common in biochars that may not be detected using typical GC–MS methods, including volatile fatty acids (VFAs) that are the main constituents of wood vinegar (also called pyroligneous acid) produced early in pyrolysis. The large number of organic compounds produced by pyrolysis and potentially sorbed by biochars complicates elucidation of the specific compounds and mechanisms involved. However, a smaller number of compounds have been detected in aqueous extracts of biochar, in particular VFAs, including acetic, propionic, butyric, valeric, and caproic acids (Rombolà et al. 2015; Gale et al. 2016; Gezahegn et al. 2021, das Graças Souza et al. 2023). VFAs have previously been found to show acute phytotoxicity at low concentrations in other contexts (e.g., Lynch 1977, 1978; Tiilikkala et al. 2010; Himanen et al. 2012). Rombolà et al. (2015) noted high concentrations of VFAs in poultry litter biochar and suggested that these contributed to inhibition of seed germination in laboratory bioassays; fast pyrolysis biochars also appear to characteristically have high concentrations of phytotoxic VFAs (Gezahegn et al. 2021).

In contrast to the generalization that VFAs are phytotoxic, it has also been suggested that wood vinegar constituents may be “hormetic”, acting to increase plant growth at low concentrations (Mu et al. 2003, 2004; Agoncillo 2018). Distinguishing between these contradictory ideas, and identifying which compounds are the main determinants of either phytotoxic or hormetic responses, demands that candidate compounds be systematically screened, preferably using formal dose–response models. Phytotoxicity of some mobile organic compounds found on biochars have been evaluated (e.g., Reynolds 1977, 1978), including studies on phytotoxicity of the most common VFAs (acetic, propionic, and butyric acids: Lynch 1977, 1978; Rao and Mikkelsen 1977; Ulbright et al. 1982a, b; Himanen et al. 2012). However, targeted work evaluating phytotoxicity of specific condensed compounds present on biochar is very limited (e.g., Bargman et al. 2013; Gezahegn et al. 2021; Shen et al. 2022). With few exceptions (Himanen et al. 2012) available estimates of phytotoxicity (i.e., ED50 values) of relevant compounds are not based on formal dose–response models. Explicit tests for possible hormetic effects have also not been conducted.

In the present study we address this research gap by assessing the relative phytotoxicity of a set of chemical compounds commonly detected in aqueous extracts of wood-feedstock biochars using bioassays of seed germination and seedling development. Prior studies have detected a relatively limited number of compounds consistently present (e,g., Spokas et al. 2011; Gale et al. 2016; Gezahegn et al. 2021, das Graças Souza et al. 2023), enhancing the feasibility of determining which of these common compounds may be responsible for toxicity effects. We address the following questions: (1) Of compounds commonly identified in aqueous biochar extracts, which are the most phytotoxic (as quantified by ED50 values for seedling germination and early development)? (2) Is there evidence for hormetic effects of any of these compounds? (3) How do target plant species vary in terms of phytotoxic (or hormetic) responses to compounds? (4) Which aspects of seedling development are most sensitive to these compounds?

## Materials and methods

### Biochars analyzed and leachate production

Biochars used were chosen on the basis of availability of detailed information on feedstocks and pyrolysis conditions. Samples analyzed included eight biochars produced using a lab-scale pyrolysis system, four using a rotating drum pyrolysis system, and one from an industrial-scale augur pyrolysis system, all with monitored temperature and residence times (Table 1). To produce leachates a homogenized 0.5-g sample from each biochar type was placed in 25 mL of deionized water for 24 h on a rotary shaking table and filtered with Whatman Grade #1 filter paper prior to analysis, replicating methods used in prior studies (Gezahegn et al. 2021).

**Table 1:** Biochars analyzed in GC–MS survey of mobile organic compounds

Feedstock	Pyrolysis system	Temp. (°C)	Residence time (min.)	References
Sugar maple^a^	lab	550	1	Gezahegn et al. (2021)
Sugar maple^a^	lab	550	5	Gezahegn et al. (2021)
Sugar maple^a^	lab	500	60	Gezahegn et al. (2021)
Sugar maple^a^	lab	700	60	Gezahegn et al. 2021
Big-tooth aspen^b^	lab	550	1	Gezahegn et al. (2021)
Big-tooth aspen^b^	lab	550	5	Gezahegn et al. (2021)
American beech^c^	lab	300	60	Gezahegn et al. (2021)
American beech^c^	lab	600	60	Gezahegn et al. (2021)
Coconut^d^ coir	batch	350	210	Thomas et al. (2019)
Sugar maple^a^	batch	374	90	Sackett et al. (2015)
Black spruce^e^/white spruce^f^	batch	328	90	Sackett et al. (2015)
Sugar maple^a^	batch	525	120	NA
Sugar maple^a^*/*Yellow birch^g^	augur	600	30	Gale et al. (2016)

Feedstock consisted of coarse wood sawdust unless otherwise indicated. Temp. is the highest recorded treatment temperature. Additional information on biochar properties can be found in references cited (NA indicates not applicable).

^a^*Acer saccharum* Marshall

^b^*Populus grandidentata* Michaux

^c^*Fagus grandifolia* Ehrh

^d^*Cocos nucifera* L

^e^*Picea glauca* (Moench) Voss

^f^*Picea mariana* (Mill.) Britton, Sterns & Poggenburg

^g^*Betula alleghaniensis* Britt

### GC–MS analysis of leachable organic compounds in biochars

Methods for characterization of mobile organic compounds present in aqueous biochar extracts replicated Rombolà et al. (2015) and Gale et al. (2016), with the use of direct-injection solid-phase microextraction (DI-SPME) enabling the detection of VFAs. SPME–GC–MS analyses were conducted at the Teaching and Research in Analytical Chemistry and Environmental Sciences (TRACES) Centre, University of Toronto, Scarborough, and the Analest facility, University of Toronto. DI-SPME analysis of leachates was performed by spiking 3 mL of deionized water leachates with 2.5 mL of 1 ppm O-eugenol, 1.0 mL of 2 M KH_2_PO_4_ buffer, and 2.5 mL of 2-ethylbutyric acid as internal standards, and then placing samples into 10-mL vials. A Carboxen-PDSM fiber was inserted into the solution under magnetic stirring for 30 min at 250 °C. GC analysis utilized a DB-WAX column (dimensions: 20 m long, 0.20 µm wide, 0.1 mm i.d.) with the following thermal sequence: 80 °C for 4 min, followed by 10 °C min^−1^ increase to 250 °C for a total run time of 28 min. MS analysis was performed on a 5975C GC–MS instrument (Agilent Technologies, Santa Clara, CA, USA) with peak identification based on the NIST-08 reference spectral library. Quantification of analyte concentrations was not attempted (cf. Spokas et al. 2011; Gale et al. 2016); concentrations of VFAs for a subset of biochars analyzed are reported elsewhere (Gezahegn et al. 2021).

### Phytotoxicity assays

Phytotoxicity assays were conducted on the most common compounds found in our analyses as well as those found in prior analyses (in particular Spokas et al. 2011). Reagent-grade (≥ 99% purity) chemicals used in assays were obtained from commercial sources. Specific sources were as follows (common name followed by IUPAC name, supplier, and CAS number): acetic acid (Labchem 64-19-7; Labchem Inc., Zelienople, PA, USA), propionic acid (propanoic acid: Alfa Aesar 79-09-4; Thermo Fisher Inc., Waltham, MA, USA), butyric acid (butanoic acid: Sigma-Aldrich (S-A) 107-92-6; Sigma-Aldrich Inc., St. Louis, MO, USA), valeric acid (pentanoic acid: S-A 109-52-4), benzoic acid (Alfa Aesar 65-85-0), phenol (Labchem 108-95-2), caproic acid (hexanoic acid: S-A 142-62-1), 2-ethylbutyric acid (2-ethylbutanoic acid: S-A 88-09-5), acetone (propan-2-one: Caledon 67-64-1; Caledon Laboratory Chemicals Inc., Georgetown, ON, Canada), benzene (S-A 71-43-2), toluene (methylbenzene: Caledon 9201-2-10), butanone or methyl ethyl ketone (butan-2-one: Caledon 7401-2), methyl salicylate (methyl 2-hydroxybenzoate: S-A 119-36-8), and 2,4-di-tert-butylphenol (S-A 96-76-4). Dilution series were made from a stock 0.1 mol L^−1^ solution for each compound. Concentrations assessed were 0.01, 0.03, 0.1, 0.3, 1, 3, 10, 30, and 100 mmol L^−1^, with concentrations omitted if above solubility. In some cases of high or low solubility, the dilution series was extended (a full list of concentrations used for each chemical is given in Supplemental Table S1). Deionized water controls were also assessed for each trial.

Experimental replicates consisted of 90-mm-diameter Petri dishes containing Whatman grade #1 filter paper, with 5 mL of a given treatment solution (as listed in Supplementary Table 1) added to each replicate. In the first set of experiments three target plant species were assessed: lettuce (*Lactuca sativa* L.), radish (*Raphinus raphanistrum* L.), and annual ryegrass (*Lolium multiflorum* Lam.) for 12 of the compounds listed above. Twenty-five seeds of a given species were added to each replicate, with 3 replicates per species per treatment. After 7 days, each replicate was assessed for germination rate (seeds were scored as germinated if the radicle was fully emerged from seed coat), cotyledon formation (scored as formed if at least one cotyledon was fully formed), and radicle length (measured from root tip to seed coat to the nearest 1 mm for a randomly determined set of 3 seeds per replicate Petri dish). In the case of radish, radicle length was measured after 3 days due to rapid growth. Germination rate and cotyledon formation were expressed as proportions, and the mean radicle length per replicate Petri dish calculated prior to analysis. The experiment was conducted in sequential batches in a controlled environment: the average temperature was 23 °C with 7W incandescent supplemental lights providing a 12-h photoperiod. Petri dish lids served to maintain high humidity.

The first set of experiments indicated possible hormetic effects of a subset of VFAs on radicle extension growth of the smaller-seeded test species. To enhance statistical power to detect effects, we conducted a second set of experiments focused on VFAs and using a higher number of replicates (10 replicate deionized water controls and 5 of each treatment) and smaller number of seeds per Petri dish (10) to ensure that all radicles could be measured with no possible seedling selection bias. The second set of experiments also included two additional VFAs commonly detected on biochars (caproic acid and 2-ethylbutyric acid) and substituted a smaller-seeded test species (basil: *Ocimum basilicum* L.) for radish. Average seed masses (fresh weight) for each species were determined using a semi-microbalance.

### Statistical analysis

Dose–response models (Ritz 2010) were used to assess responses of seed germination and early plant development to treatments. For each response variable (percent germination, percent cotyledon formation, and radicle length) we compared a set of alternative dose–response models based on minimum AIC (Akaike Information Criterion) values. The models compared included 3- and 4-parameter logistic and log-logistic functions, defined as follows:

1 $$f\left( {x; \, b,c,d,e} \right) = c + \left( {d{-}c} \right)/(1 + exp(b\left( x \right){-}e))$$

2 $$f\left( {x; \, b,c,d,e} \right) = c + \left( {d{-}c} \right)/(1 + exp(b(ln\left( x \right){-}ln(e))))$$

where *f(x)* is the response variable, and *x* is dosage. The parameter *b* reflects the steepness of the dose–response function, *c* is the lower asymptote at high concentrations, *d* is the upper asymptote value at low concentrations, and *e* is the dose corresponding to the ED50 value (the point at which 50% of the response is detected). In both cases the 3-parameter model sets c = 0. We examined relationships between ED50 values among the different response variables examined (percent germination, percent cotyledon formation among germinated seedlings, and radicle length), using linear regression on log-transformed ED50 values.

The model of Cedergreen et al. (2005) was initially used to test dose–response curves for an initial increase in plant performance with concentration (i.e., hormesis). The function used was:

3 $$f(x; \, b,c,d,e,f,a) = c + (d{-}c + f \, exp( - 1/x^{a} )/\left( {1 + exp\left( {b\left( {ln\left( x \right){-}ln\left( e \right)} \right)} \right)} \right)$$

where f describes the magnitude of the hormetic effect, and *α* is a constant describing the rate of increase of the hormetic peak with dosage; in this case the interpretation of other constants differs from Eqs. (1) and (2) (see Cedergreen et al. 2005 for details). A test of the null hypothesis *f* = 0 was treated as a test for hormetic effects (Cedergren et al., 2005). Parameter estimates for Eq. (2) were used to initialize non-linear curve-fitting for Eq. (3), and an *α* constant of 0.5 was assumed. Analyses were conducted in the statistical programming environment R (R Core Team 2023), using the “drc” package to estimate dose–response functions (Ritz and Streibig 2005). 

The Cedergreen et al. (2005) hormesis test may have low statistical power as it depends on the fit of the overall function as well as the existence of a peak at intermediate concentrations. We therefore conducted a second set of tests for hormesis using post-hoc comparisons to the control based on the Dunnett test (Jaki and Hothorn 2013). This test was used only for radicle length effects and excluded replicates with no seed germination. ANOVA significance was checked prior to post-hoc analysis, as were assumptions of normality and equal variance of residuals (evaluated graphically using diagnostic plots). In addition, to provide a global test for hormesis of VFAs pooling all species and trials, we conducted a random-effects meta-analysis, which was called for since the effect size was expected to vary among species and potentially among trials (Borenstein et al. 2010). This analysis focused on comparisons between deionized water controls and the lowest concentrations of VFAs assessed (0.01 and 0.03 mmol L^−1^). We used the response ratio statistic (R = ln(X_t_/X_c_) as the effect size statistic, where X_t_ is the treatment mean and X_c_ is the control mean; pooled R values were weighted by the inverse of sampling variance. Statistical tests for effects were based on a normal approximation and the Q-test for heterogeneity, as implemented in the R package “metafor” (Viechtbauer 2010). R values were used to compute the percent change as 100 × (exp(R) − 1).

## Results

### GC–MS analysis of aqueous extracts and volatiles from biochars

Based on comparisons to the NIST-08 library, we identified 151 organic compounds derived from the 13 biochars tested (Supplemental Table S2). Biochar leachates commonly contained volatile fatty acids including acetic (100% of leachates), valeric (100%), caproic (92%), 2-ethylbutyric (86%), propionic (38%), and benzoic acids (31%) (Table 2). Butyric acid was not detected in leachates but was detected in 31% of solid-phase analyses. Other compounds detected in > 50% of tested biochars included 2,4-Di-tert-butylphenol, methyl salicylate, diethyl acetic acid, and 4-hydroxy-3,5-dimethoxy benzaldehyde, diethyl phthalate, and *p*-pentylacetophenone. Commonly detected compounds identified that are consistent with prior publications include benzaldehyde and 2-hexene (Spokas et al. 2011), pyridine (Yuan et al. 2017), and 2,4-di-tert-butylphenol (Gale et al. 2016). The only detected polycyclic aromatic hydrocarbon (PAH) of toxicity concern (included on either the EPA 16-compound list or an extended 40-compound list: Andersson and Achten 2015) was fluorene, which was detected in 36% of the biochars sampled. We did not detect some of the common compounds found in prior surveys (Spokas et al. 2011, 2012); however, those found in 60% or more of samples from these prior studies (i.e., acetone, benzene, toluene, and methyl ethyl ketone) were also included in the phytotoxicity assays.

**Table 2:** Mobile organic compounds detected in at least 50% of the biochars tested in aqueous extracts or solid phase by direct injection GC–MS analysis (IUPAC names followed by common names were applicable)

Compound	Frequency
*Acetic acid	13/13
*Valeric acid (Pentanoic acid)	13/13
*2,4-Di-tert-butylphenol	13/13
*Methyl salicylate (Oil of wintergreen)	12/13
*Caproic acid (Hexanoic acid)	12/13
*2-Ethylbutyric acid	11/13
Benzaldehyde, 4-hydroxy-3,5-dimethoxy-	10/13
Diethyl phthalate	6/13
*p-Pentylacetophenone	6/13

A full list of identified compounds is given in supplemental Table 1. Compounds detected in aqueous biochar leachates are marked “*”

### Phytotoxicity assays

**Fig. 1:**
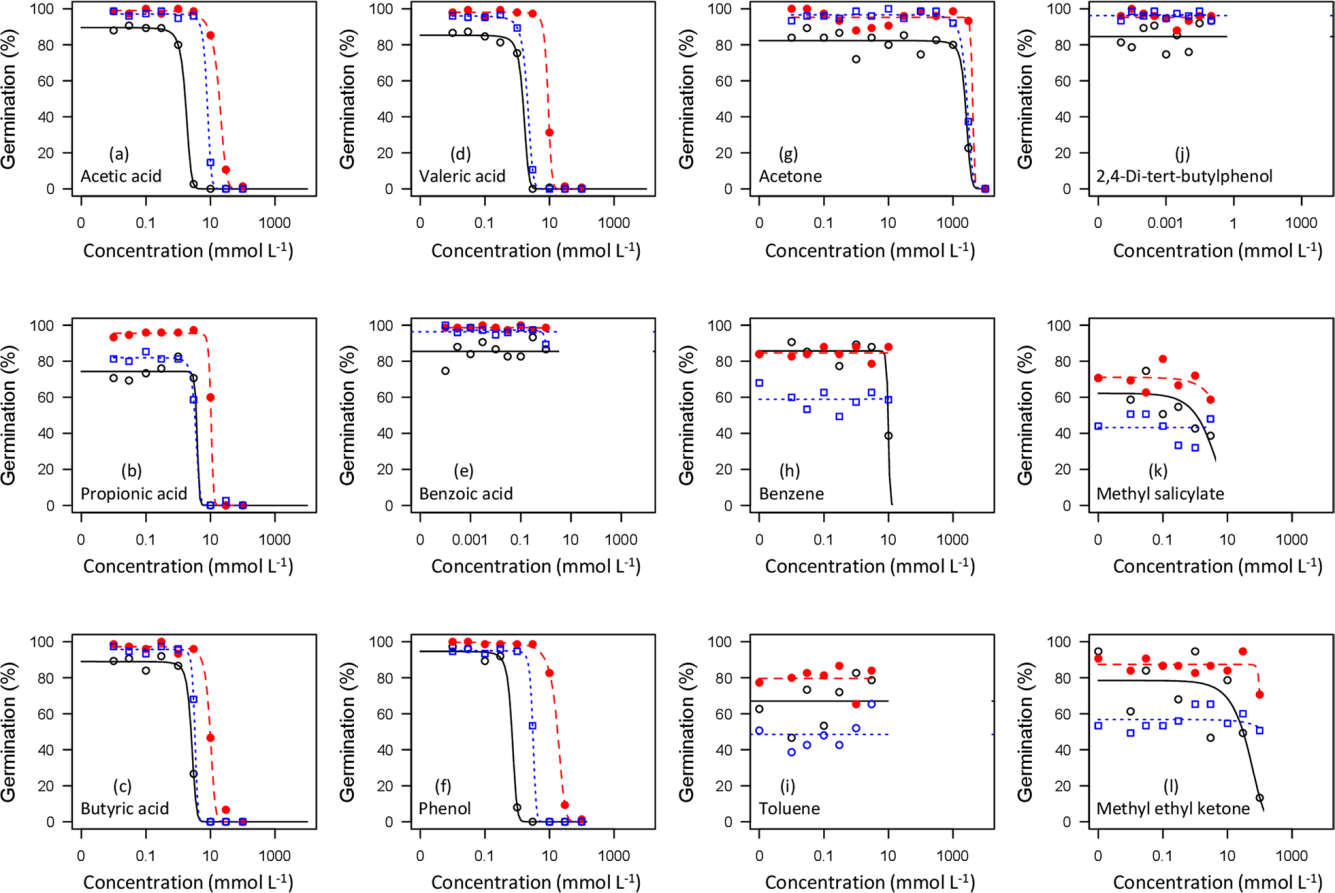
Percent germination in seeds of three agricultural species as a function of concentrations of 12 mobile organic compounds identified in biochar leachates. Symbols represent the mean of 3 replicates: open (black) circles with solid line are lettuce; open (blue) squares with dotted line are ryegrass, and closed (red) circles with dashed line are radish. Fitted curves are for the logistic model (Eq. 1) with the upper asymptote parameter (c) fixed to zero

In the case of VFAs with high solubility (acetic acid, propionic acid, butyric acid, and valeric acid), as well as phenol (also an important wood vinegar constituent) the dilution series of chemicals showed clear phytotoxic effects on germination rates, with no germination at the highest concentrations examined, and a distinct threshold dose consistent with fitted dose–response relationships (Fig. 1a–d, f). In contrast, for most non-VFA compounds tested (benzene, toluene, methyl ethyl ketone, methyl salicylate, benzoic acid, and 2,4-di-tert-butylphenol) little or no germination response was observed even in near-saturated solutions (Fig. 1e, h–l). ED50 values for germination ranged from 1.5–16.7 mmol L^−1^ for VFAs (acetic acid, propionic acid, butyric acid, and valeric acid), and 0.6–5.8 mmol L^−1^ for phenol (Table 3). In contrast, germination-based ED50 values for other compounds, where estimable, were 10–1000-fold higher, ranging from ~ 10–6000 mmol L^−1^ (Table 3).

**Table 3:** Estimated ED50 values (± 95% C.I.) for germination, radicle extension, and cotyledon formation for major biochar aqueous extract constituents in four target plant species (combining results from experiments 1 and 2)

Chemical	Species	Germination ED50 (mmol L^−1^)		P	Radicle extension ED50 (mmol L^−1^)		P	Cotyledon formation ED50 (mmol L^−1^)		P
Acetic acid	Lettuce	1.52	± 0.18	< 0.001	1.00	± 0.00	< 0.001	1.33	± 2.01	0.516
	Ryegrass	7.16	± 0.85	< 0.001	3.36	± 0.56	< 0.001	3.29	± 4.12	0.004
	Radish	16.72	± 0.49	< 0.001	3.08	± 0.36	< 0.001	3.82	± 24.71	0.879
	Basil	3.73	± 0.99	< 0.001	1.76	± 0.26	< 0.001	1.86	± 0.48	< 0.001
Propionic acid	Lettuce	3.87	± 0.09*	< 0.001	0.91	± 0.10	< 0.001	1.35	± 0.15	< 0.001
	Ryegrass	3.50	± 0.19*	< 0.001	1.57	± 0.25	< 0.001	4.79	± 5.19	0.365
	Radish	10.76	± 4.26	0.019	7.02	± 4.45	0.127	4.45	± 3.55	0.222
	Basil	2.58	± 0.44	< 0.001	0.58	± 0.06	< 0.001	1.19	± 0.17	< 0.001
Butyric acid	Lettuce	2.44	± 0.35	< 0.001	0.87	± 0.32	0.013	1.23	± 1.11	0.278
	Ryegrass	3.38	± 1.45	0.029	1.24	± 0.19	< 0.001	3.73	± 2.09	0.088
	Radish	9.79	± 0.31	< 0.001	2.42	± 0.58	< 0.001	8.20	± 2.47	0.003
	Basil	7.16	± 3.03*	0.022	0.81	± 0.10	< 0.001	1.77	± 0.67	0.010
Valeric acid	Lettuce	1.59	± 0.08*	< 0.001	0.93	± 0.16	< 0.001	1.18	± 3.09	0.703
	Ryegrass	1.85	± 0.08	< 0.001	0.97	± 0.08	< 0.001	3.36	± 2.32	0.154
	Radish	8.42	± 0.93	< 0.001	2.75	± 0.75	0.001	6.64	± 0.78	< 0.001
	Basil	2.07	± 0.51*	< 0.001	0.52	± 0.04	< 0.001	1.06	± 0.29	< 0.001
Caproic acid	Lettuce	1.46	± 0.53	0.008	0.33	± 0.04	< 0.001	1.02	± 0.12	< 0.001
	Ryegrass	2.86	± 0.32	< 0.001	1.39	± 0.15	< 0.001	2.44	± 1.68	0.152
	Basil	4.00	± 1.04	< 0.001	0.66	± 0.07	< 0.001	1.84	± 0.36	< 0.001
2-ethylbutyric acid	Lettuce	0.741	± 1.161	< 0.001	0.203	± 0.039	< 0.001	0.658	± 0.170	< 0.001
	Ryegrass	1.084	± 0.292	< 0.001	0.114	± 0.013	< 0.001	0.887	± 0.247	< 0.001
	Radish	0.708	± 0.120*	< 0.001	0.085	± 0.008	< 0.001	0.371	± 0.090	< 0.001
Acetone	Lettuce	2,365	± 513	< 0.001	269	± 123	0.035	513	± 189	0.010
	Ryegrass	2,597	± 140	< 0.001	2,449	± 437	< 0.001	4,902	± 197	< 0.001
	Radish	4,632	± 3,890	0.242	2,399	± 854	0.008	5,606	± 98	< 0.001
Phenol	Lettuce	0.62	± 0.05	< 0.001	0.44	± 0.32	0.191	2.12	± 3.66	0.878
	Ryegrass	3.14	± 0.19	< 0.001	2.14	± 0.27	< 0.001	3.83	± 1.33	0.008
	Radish	5.80	± 0.68	< 0.001	1.25	± 0.32	0.001	2.13	± 0.15	< 0.001
Benzene	Lettuce	9.74	± 1.03	< 0.001	NA	NA	NA	9.67	± 1.80	0.010
	Ryegrass	NA	NA	NA	NA	NA	NA	NA	NA	NA
	Radish	NA	NA	NA	NA	NA	NA	NA	NA	NA
Methyl ethyl ketone	Lettuce	42.0	± 11.7	< 0.001	77.8	± 30.9	0.018	39.3	± 10.1	< 0.001
	Ryegrass	NA	NA	NA	185.4	± 88.1	0.045	NA	NA	NA
	Radish	137.0	± 57.8	0.025	57.8	± 62. 7	0.037	NA	NA	NA

**Fig. 2:**
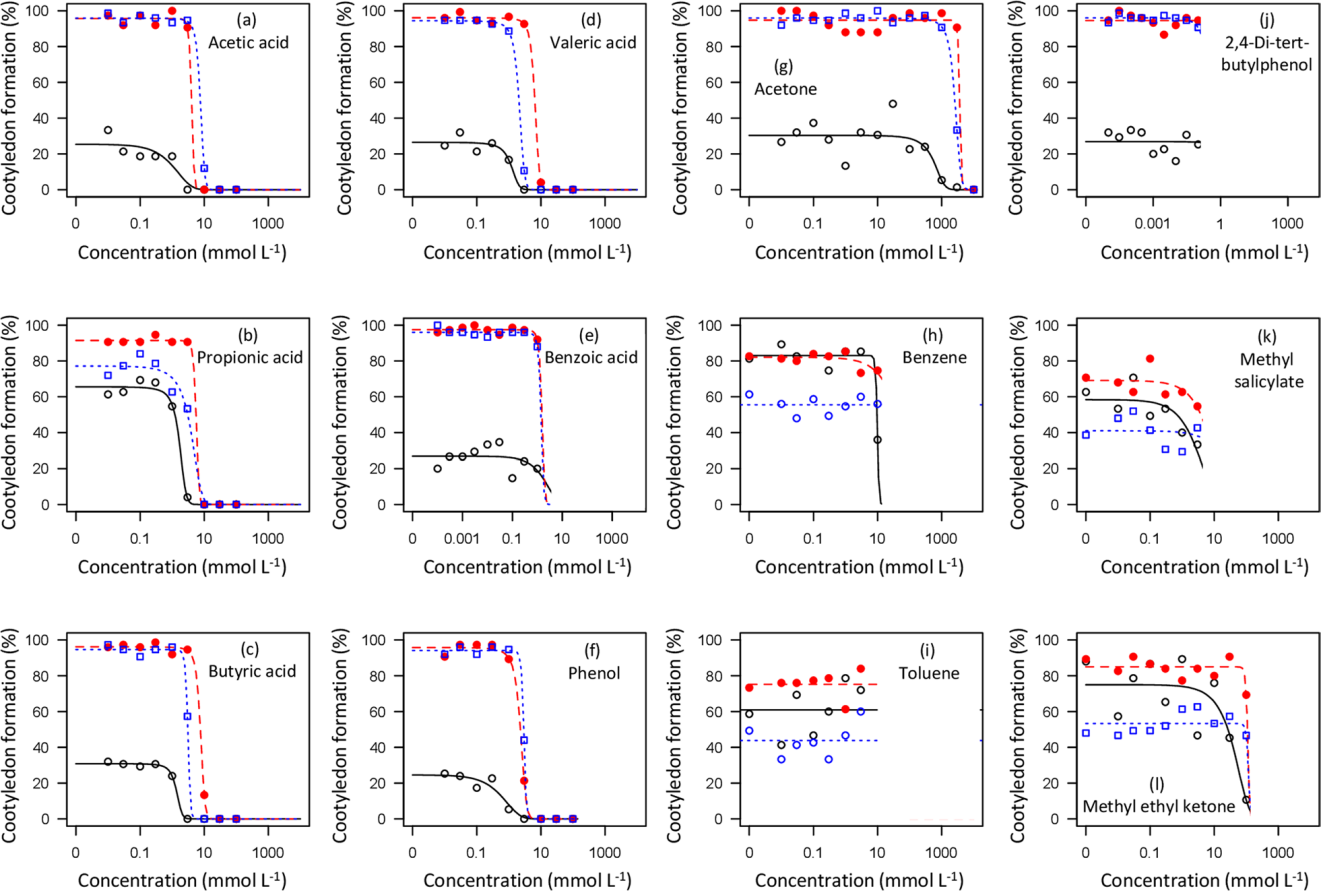
Percent of germinated seeds forming cotyledons in seeds of three agricultural species as a function of concentrations of 12 mobile organic compounds identified in biochar leachates. Symbols represent the mean of 3 replicates: open (black) circles with solid line are lettuce; open (blue) squares with dotted line are ryegrass and closed (red) circles with dashed line are radish. Fitted curves are for the logistic model (Eq. 1) with the upper asymptote parameter (c) fixed to zero

**Fig. 3:**
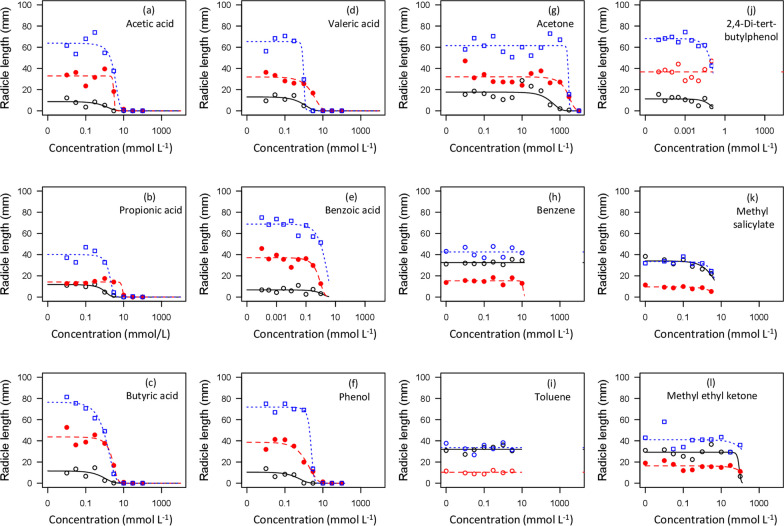
Radicle extension growth in seeds of three agricultural species as a function of concentrations of 12 mobile organic compounds identified in biochar leachates. Symbols represent the mean of 3 replicates: open (black) circles with solid line are lettuce; open (blue) squares with dotted line are ryegrass and closed (red) circles with dashed line are radish. Fitted curves are for the logistic model (Eq. 1) with the upper asymptote parameter (c) fixed to zero

**Fig. 4:**
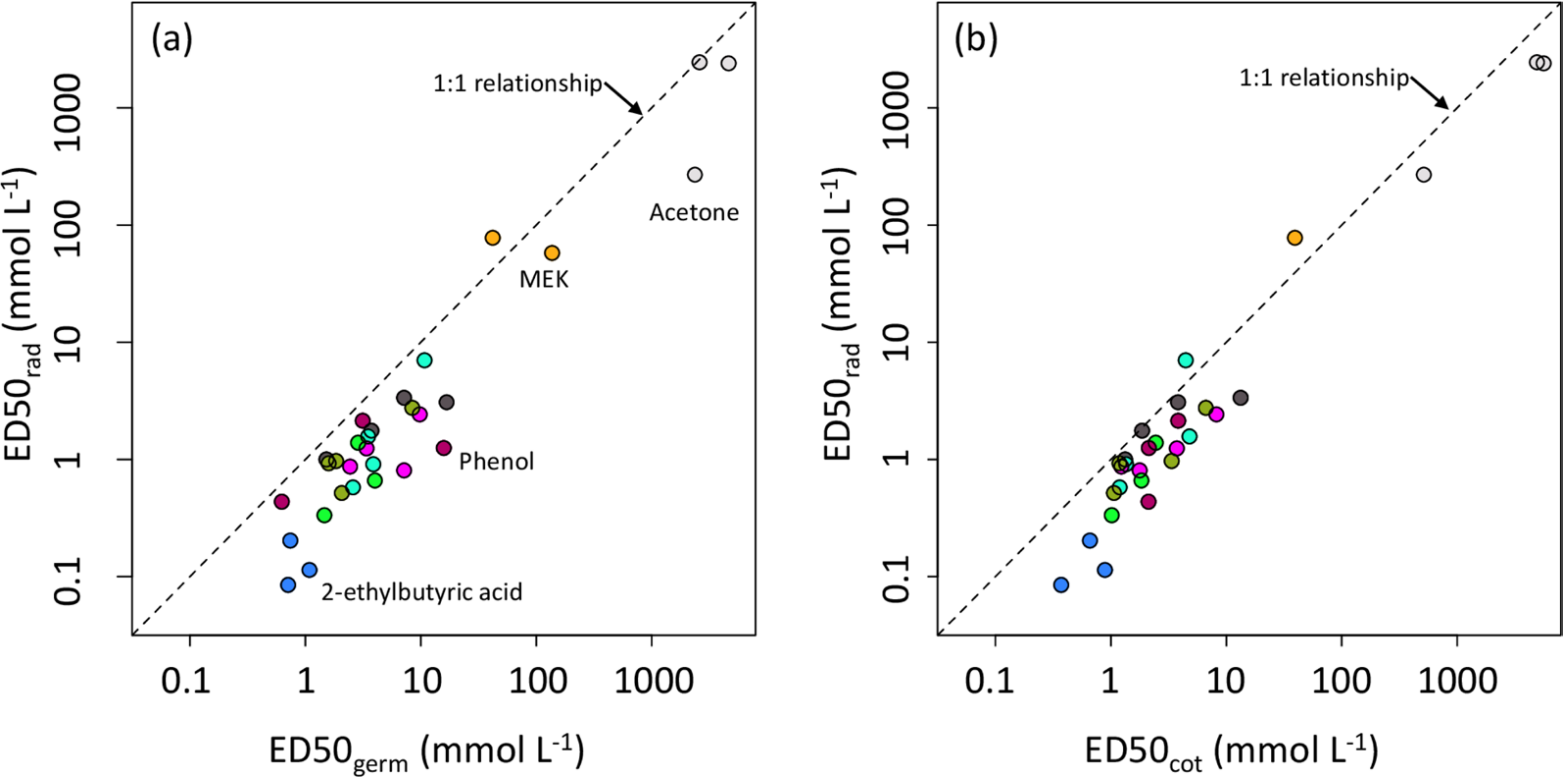
ED50 values for **a** radicle extension and **b** cotyledon formation as a function of ED50 for germination (combining data from experiments 1 and 2 as shown in Table 2). Colors correspond to chemical compounds tested: grey is phenol; orange is methyl ethyl ketone; brown is phenol; dark blue is 2-ethylbutyric acid; other colors are straight-chain VFAs. The dashed line shows a 1:1 relationship

All values listed are based on a log-logistic dose–response model as shown in Figs. 2, 3, 4, with exceptions (*) based on the logistic dose–response model where this showed a lower AIC value. Cases in which ED50 values were not estimable are marked “NA” and compounds tested that did not show significant ED50 values in any test species are excluded

Dose–response patterns for radicle extension growth and cotyledon formation also showed clear dose–response thresholds for VFAs and phenol (Figs. 3–4, Table 3). In addition, benzoic acid and 2,4-di-tert-butylphenol did have detectable negative effects on seedling development in some cases, based on linear regressions of seedling performance versus log-transformed concentrations. Significant negative effects of benzoic acid on radicle extension were found for radish (*r* = − 0.565; *p* = 0.002) and ryegrass (*r* = − 0.590; *p* = 0.001), but not lettuce (*r* = − 0.165; *p* = 0.411); no effects on cotyledon development were observed (*p* > 0.05). Negative effects of 2,4-di-tert-butylphenol on radicle extension were found for lettuce (*r* = − 0.454; *p* = 0.017) and ryegrass (*r* = − 0.558; *p* = 0.003), but not radish (*r* = 0.056; *p* = 0.780); a negative effect of 2,4-di-tert-butylphenol on cotyledon development was also observed for ryegrass (*r* = − 0.425; *p* = 0.027), but not the other species (*p* > 0.05). ED50 values were not estimable in any of these cases as the declines in seedling development were roughly linear with respect to log-transformed concentration values, and effects were less than a 50% reduction (Figs. 3, 4).

Estimated ED50 values for radicle extension growth (ED50_rad_) and cotyledon formation (ED50_cot_) were closely correlated with those for germination (ED50_germ_) (Fig. 4). ED50 values spanned 5 orders of magnitude; therefore, values were log-transformed prior to analysis. Correlations for log-transformed values were 0.959 for ED50_germ_ vs. ED50_rad_, 0.951 for ED50_germ_ vs. ED50_cot_, and 0.973 for ED50_cot_ vs. ED50_rad_ (all significant at *p* < 0.001). ED50_rad_ values were consistently lower than ED50_germ_ values (*p* < 0.001; paired t-test of log-transformed values), or ED50_cot_, (*p* < 0.001); ED50_germ_ were also somewhat lower than ED50_cot_ (*p* = 0.013).

The relative sensitivity of species to chemical exposure was generally consistent for all chemicals and all measures of seedling development, with lettuce the most sensitive, followed by ryegrass and then radish (considering data from experiment 1). ED50 values followed this order in 6 of 7 cases for germination, and 3 of 7 cases each for cotyledon development and germination; in all cases lettuce showed a lower ED50 value than radish (Table 3). To test statistically for this pattern, we conducted ANOVAs of log-transformed ED50 values from experiment 1 with chemical and species as independent variables (with no interaction term). The main effect term for chemical was highly significant in all cases (*p* < 0.001); the term for species was likewise significant (*p* < 0.001 for germination and cotyledon formation; *p* = 0.023 for radicle extension).

### Tests for hormetic effects

Hormetic effects correspond to a peak value in plant developmental response at some intermediate concentration: i.e., show a unimodal response pattern (Cedergreen et al. 2005). For both germination and cotyledon formation, the constant *f* in the Cedergreen et al. model (Eq. 3) was not significant in any of the modeled dose–response functions examined and the Cedergreen et al. model also showed higher AIC values than monotonic models. In contrast to results for germination and cotyledon formation, radicle length growth did exhibit apparent peak values above controls in several cases, in particular for valeric acid and acetic acid in the case of ryegrass (Fig. 3). Nevertheless, the constant *f* in the Cedergreen et al. model was only marginally significant in these cases.

**Fig. 5:**
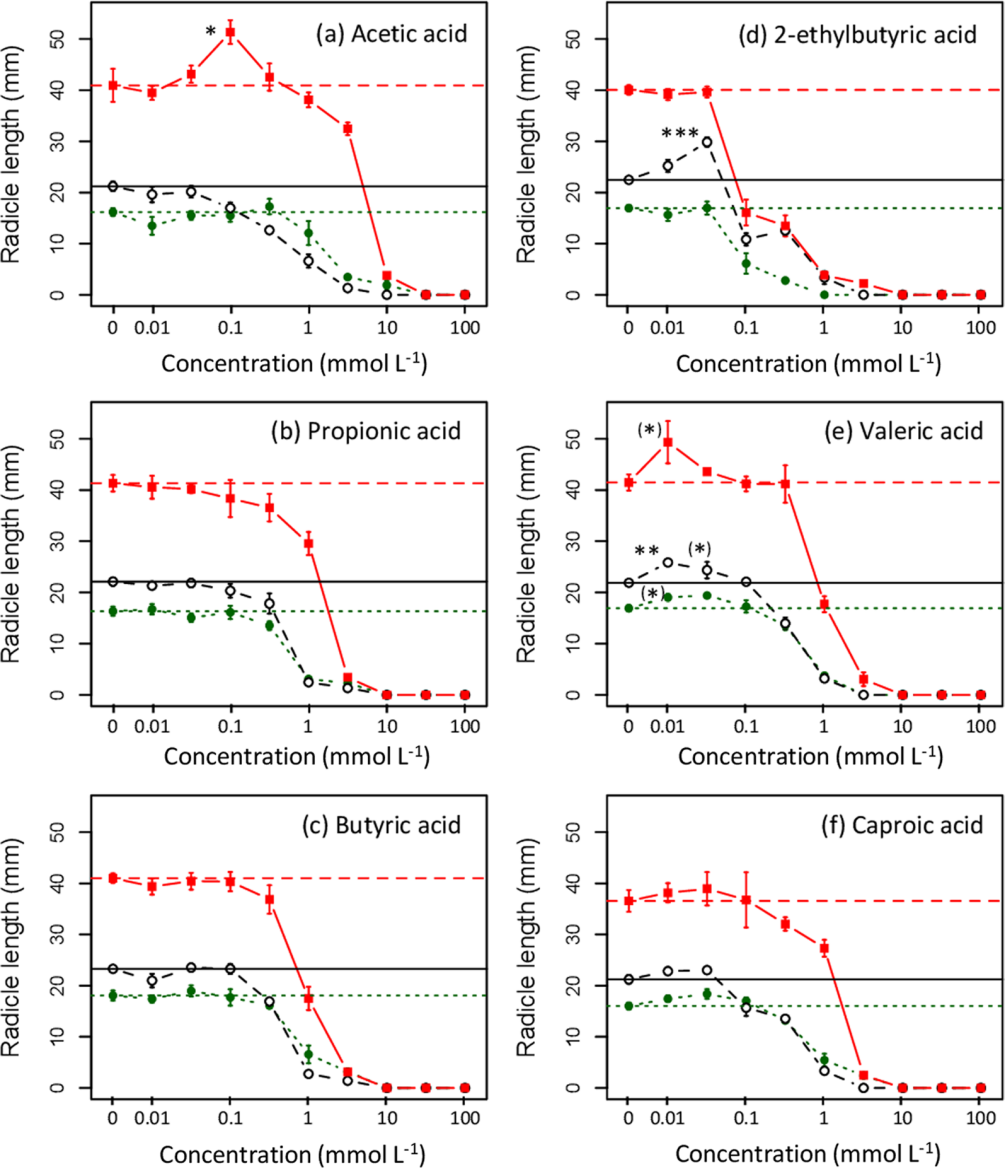
Radicle extension growth of three species (lettuce, ryegrass, and basil) as a function of concentrations of 6 VFAs identified in aqueous biochar leachates. Symbols represent the mean of 5 replicates (10 in the case of deionized water controls): open (black) circles with solid line are lettuce; closed (red) squares with dashed line are lettuce and closed (green) circles with dotted line are basil. Treatments showing a significant increase relative to deionized water controls (based on a Dunnett post-hoc test) are marked as: (*) 0.1 < *p* < 0.05; *p < 0.05; **p < 0.01; ***p < 0.001

Given these ambiguous results, experiment 2 was conducted to increase statistical power to detect possible hormetic effects of VFAs on radicle extension, and to include additional VFAs common in biochar leachates (caproic acid and 2-ethylbutryic acid). Consistent with observations in experiment 1, there was evidence for hormesis in valeric acid and acetic acid, with a significant positive effect on lettuce at 0.01 mmol L^−1^ for valeric acid, and on ryegrass at 0.1 mmol L^−1^ for acetic acid compared to controls (Fig. 5a, e). In addition, 2-ethylbutyric acid resulted in a significant increase in radicle growth in lettuce at 0.03 mmol L^−1^ (Fig. 5d). Neither propionic nor butyric acid showed any evidence for hormetic effects (Fig. 5b, c); however, results for caproic acid suggest a slight increase in radicle extension at 0.01–0.03 mmol L^−1^ concentrations in all three test species (Fig. 5f), though this pattern was not judged significant.

**Fig. 6:**
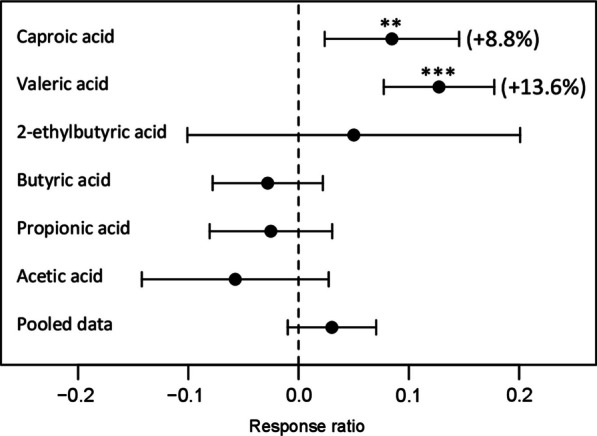
Meta-analysis of species-pooled effects of low concentrations (0.01–0.03 mmol L^−1^) of VFAs on radicle extension growth, based on results of experiment 2. The effect size is measured as the log response ratio and is plotted ± 95% confidence limits. Statistical significance: **p < 0.01; ***p < 0.001

A combined analysis (i.e., pooling experimental trials and species) for effects of low concentrations (0.01–0.03 mmol L^−1^) of VFAs on radicle extension growth was conducted using a random-effects meta-analysis (Borenstein et al. 2010) for experiment 2 data (Fig. 6). The overall test for heterogeneity of effects among compounds was significant (*Q*_*17*_ = 50.65; *p* < 0.001). Significant positive effects were detected in the case of valeric acid (*z* = 4.979; *p* < 0.001) and caproic acid (*z* = 2.725; *p* = 0.006), but not other compounds (Fig. 6).

## Discussion

The present study builds on extensive evidence that biochars commonly sorb organic compounds, some of which can have phytotoxic effects. Among the most common compounds detected in aqueous biochar extracts, it is clear that volatile fatty acids (VFAs) are among the most common chemicals present, and also have low thresholds for acute toxic effects on seed germination and early seedling development. This is consistent with prior suggestions that VFAs are the most common mobile organic compounds in biochars that elicit acute phytotoxicity (Rombolà et al. 2015; Gale et al. 2016; Gezahegn et al. 2021). However, our analyses also indicate that certain VFAs present in biochars elicit hormetic effects, inducing small to moderate increases in radicle extension growth at low concentrations. Such effects might offset phytotoxicity and could potentially account for positive effects of biochar leachates on early seedling development that have been reported in some prior studies.

### Phytotoxicity

Higher plants are only infrequently used in standard toxicity testing (Wang 1991); for example, higher plants make up only ~ 2.6% of the records for toxicological testing of organic chemicals in the EPA Ecotox database (Olker et al. 2022; searched Nov. 2023). Thus, while some of the chemicals examined here are common in industrial and other settings, quantitative evaluations of higher plant toxicities are mostly lacking. Prior assessments of phytotoxicity for some VFAs are available, particularly in the context of wet rice cultivation (Lynch 1977, 1978, 1980). While formal assessments of effective dosages (Ritz 2010) appear not to have previously been published, graphical results suggest an ED50 value of ~ 10 mmol L^−1^ for acetic acid and ~ 3 mmol L^−1^ for butyric acid based on root extension of *Hordeum vulgare* (Lynch 1980, his Fig. 1). In the present study we detected ED50 values ranging from 1–15 mmol L^−1^ for acetic, propionic, and butyric acids, corresponding closely to these published data. Other wood vinegar constituents, including valeric acid, caproic acid, and phenol similarly showed toxicity thresholds in this approximate range (Figs. 2, 3, 4; Table 2). The reported ED50 values for VFAs were similar to those from a prior unpublished study that used *Lepidium sativum* and *Lolium multiflorum* as test species (Prochazka 2008).

The branched VFA 2-ethylbutyic acid stands out as the most phytotoxic of the VFAs and other compounds tested, with ED50 values as low as ~ 0.1–0.2 mmol L^−1^ for radicle extension (Fig. 4, Table 2). Toxic effects of this compound do not appear to have previously received attention; there is no record in the EPA Ecotox database (Olker et al. 2022; searched Nov. 2023) or in cited literature. 2-ethylbutyic acid was among the most common compounds detected in biochars (Table 2), as has been noted in prior studies (Rombolà et al. 2015).

Aside from VFAs, other biochar leachate constituents tested generally had much higher ED50 values, or only small effects on seedling development even at concentrations approaching saturation. ED50 values for acetone were in the range of 300–6900 mmol L^−1^, or roughly 3 orders of magnitude higher than those for VFAs. Benzoic acid, toluene, and 2,4-di-tert-butylphenol had no detectable effects on germination or cotyledon development even at concentrations approaching saturation (Figs. 2, 3). Several other compounds (benzene, methyl ethyl ketone, methyl salicylate) had detectable negative effects, but only at concentrations near saturation (Figs. 2, 3). Benzoic acid has received some prior attention as an allelochemical; we observed detectable negative effects on radicle extension (but not cotyledon development) in radish and ryegrass, consistent with prior studies (Yu and Matsui 1994; Kaur and Kaushik 2005; Fernández-Aparicio et al. 2013). Other non-VFA compounds tested (benzene, toluene, 2,4-di-tert-butylphenol, methyl ethyl ketone, methyl salicylate) exhibited either no effect or detectable but relatively small negative effects on radicle extension in one or more test species, but did not generally result in declines approaching 50%, and thus did not have estimable ED50 values.

The three test plant species in experiment 1 showed highly consistent patterns of response to tested chemicals, with lettuce generally the most sensitive, radish the least, and ryegrass intermediate (Figs. 2, 3, 4; Table 3). This ranking corresponds closely to seed size: ~ 1.3 mg, ~ 4.1 mg, and ~ 91 mg per seed for lettuce, ryegrass, and radish, respectively. It has generally been observed that smaller-seeded species are most sensitive to toxic effects on the seed germination processes (Liebman and Sundberg 2006). In addition, there were consistent patterns of response among seedling development metrics, with radicle extension generally showing lower ED50 values than germination or cotyledon development (Fig. 4). The consistency of these patterns suggests that an effective but low-cost bioassay to quantitatively estimate concentrations of phytotoxic constituents in biochar could be developed based on effects on radicle extension on a series of seeds varying in size. This strategy would be similar to protocols developed for allelochemicals (e.g., Macías et al. 2000). The most widely used bioassay for biochar phytotoxicity is based on germination responses and only measures the proportion germinated in relatively large-seeded species such as soybean and wheat (Van Zwieten et al. 2010; IBI 2015).

Prior studies on potential toxicity effects of biochar have commonly focused on polycyclic aromatic hydrocarbons (PAHs) and to some extent chlorinated dioxins and furans (as reviewed by Hale et al. 2012; Godlewska et al. 2021). This set of compounds is of particular concern in terms of effects on vertebrates (including humans), and so is the focus of biochar toxicity testing for regulatory purposes and certification (IBI 2015). However, it is less clear if these compounds play an important role in phytotoxic responses to biochar or biochar leachates. Of the 151 organic compounds identified in biochar leachates and volatiles, we detected only one on the US EPA list of 16 PAHs of toxicity concern (fluorene), which was present in about 1/3 of tested biochars. However, there is some evidence that fluorene is more phytotoxic than other PAHs (Somtrakoon and Chouychai 2013). Our data thus agree with conclusions that PAHs contribute to phytotoxicity in some biochars (Godlewska et al. 2021; Shen et al. 2022). Nevertheless, the wide prevalence, high solubility, and low toxicity thresholds of common VFAs suggest that these compounds are the main phytotoxic agents of concern in wood-feedstock biochars.

### Hormesis

The common VFAs detected in biochars correspond to the aqueous fraction products of pyrolysis commonly referred to as wood vinegar. Wood vinegar has been proposed to have hormetic effects on plants, promoting early seedling development (and potentially later plant growth) at low concentrations, while inhibiting growth at high concentrations (Mu et al. 2003, 2004; Agoncillo 2018). However, the most widely cited study (Mu et al. 2003) does not present statistical analysis of the key results on radicle extension effects, and graphically shows neutral (means ± 2 × SE overlapping control) or negative responses. The literature on VFAs has long emphasized phytotoxicity of these compounds (e.g., Prill et al. 1949; Rao and Mikkelsen 1977, Ulbright et al. 1982a, b). However, our data from experiment 2 provide clear support for hormetic effects of VFAs on seedling radicle extension, specifically for valeric acid and caproic acid, with possible species-specific hormetic effects for acetic and 2-ethylbutyric acids (Figs. 5,6).

We are not aware of any prior publications presenting strong evidence for hormetic effects of VFAs; however, an unpublished thesis interprets graphical results as possibly showing such effects (Prochazka 2008). Our results raise new questions regarding potential mechanisms for these effects and their implications for biochar processing and valorization of biochar leachates and of wood vinegar. While the evidence for hormetic effects of valeric and caproic acids is statistically strong (Fig. 6), these effects are quantitatively small (~ 5–15% increases in radicle extension). As a component of a complex mixture of compounds present in biochar leachates, this small stimulatory effect would likely be offset by negative effects of other compounds in most cases. In terms of mechanism, it has been noted that VFAs with longer aliphatic carbon chains are more lipophilic in nature, and thus more readily taken up through membranes, including root tips (e.g., Marambe et al. 1993). It is possible that within plant cells VFAs could be directly metabolized, resulting in a small increase in energy and metabolites necessary for growth.

In addition to valeric and caproic acid, it is likely that some other biochar leachate constituents present at low concentrations, such as karrikins (Kochanek et al. 2016), also have stimulatory effects on early plant development in certain plant species; there may also be synergistic effects of constituents. Our data and related studies (Rombolà et al. 2015; Gale et al. 2016; Gezahegn et al. 2021, das Graças Souza et al. 2023) indicate that VFAs and other wood vinegar constituents are an important component of leachates from un-weathered biochars; however, biochar leachates are not simply wood vinegar. Biochar leachates also contain inorganic nutrient ions and metals concentrated during pyrolysis (Gezahegn et al. 2019, 2021). Additional studies focused on chemical characterization of biochar leachates in relation to pyrolysis conditions, particle size, and feedstock are important in developing generally applicable protocols to produce “clean” biochars free from common phytotoxic compounds. A recent meta-analysis indicates that post-production heating and aeration treatments of biochar result in more positive effects on plant growth than leaching or washing treatments (Thomas 2021).

### Implications

If VFAs are the main phytotoxic component in biochars, post-production treatments could profitably focus on reducing these specific compounds. Washing or leaching of biochars can alleviate phytotoxic effects (Gale et al. 2016) but will also reduce readily leached mineral nutrients in biochars, such as potassium (Rogovska et al. 2012). Given the co-production of biochar and wood vinegar in many pyrolysis systems, addition of wood vinegar to biochar is an obvious measure to reduce the pH of highly basic biochars, with some history of use in Asia (e.g., Uddin et al. 1995; Kadota and Niimi 2004; Daosukho et al. 2012). Our results suggest that this strategy incurs a serious risk of phytotoxic effects on sensitive crops.

In conclusion, our results support the conclusion that VFAs produced during pyrolysis and sorbed by biochars are the most common compounds that contribute to cases of negative effects on early plant performance. The branched VFA 2-ethylbutyric acid is notably the most phytotoxic of all common compounds detected. Some of the VFAs (in particular valeric and caproic acids) also can produce moderate “hormetic” increases in seedling development at low concentrations (0.01–0.03 mmol L^−1^). The results suggest that preparation, post-processing, and application strategies for biochars should be conducted in a manner to produce VFA concentrations in the soil solution that fall below toxicity thresholds in the range of 0.2–20 mmol L^−1^.

### Supplementary Information

Supplementary Material 1.

Supplementary Material 2.

Supplementary Material 3.

Supplementary Material 4.

## Data Availability

Data are available as supplementary information.

## References

[CR1] Agoncillo ES (2018). Vegetable seed germination enhancement using different levels of pyroligneous acid (PA). J Biol Agric Healthcare.

[CR2] Andersson JT, Achten C (2015). Time to say goodbye to the 16 EPA PAHs? Toward an up-to-date use of PACs for environmental purposes. Polycyclic Aromat Compd.

[CR3] Bargmann I, Rillig MC, Buss W, Kruse A, Kuecke M (2013). Hydrochar and biochar effects on germination of spring barley. J Agron Crop Sci.

[CR4] Biederman LA, Harpole WS (2013). Biochar and its effects on plant productivity and nutrient cycling: a meta-analysis. GCB Bioenergy.

[CR150] Bieser JM, Thomas SC (2019). Biochar and high-carbon wood ash effects on soil and vegetation in a boreal clearcut. Can J For Res.

[CR5] Borenstein M, Hedges LV, Higgins JP, Rothstein HR (2010). A basic introduction to fixed-effect and random-effects models for meta-analysis. Res Synth Methods.

[CR7] Buss W, Mašek O (2014). Mobile compounds in biochar – a potential source of contamination – phytotoxic effects on cress seed (*Lepidium sativum*) germination. J Environ Manage.

[CR9] Buss W, Mašek O, Graham M, Wüst D (2015). Inherent compounds in biochar – their content, composition and potential toxic effects. J Environ Manage.

[CR11] Cedergreen N, Ritz C, Streibig JC (2005). Improved empirical models describing hormesis. Environ Toxic Chem.

[CR12] Chia CH, Downie A, Munroe P (2015) Characteristics of biochar: physical and structural properties. In: Lehmann J, Joseph S (eds) Biochar for Environmental Management: Science, Technology and Implementation, pp 89–109

[CR13] Clough TJ, Condron LM, Kammann C, Müller C (2013). A review of biochar and soil nitrogen dynamics. Agronomy.

[CR16] Dai Y, Zheng H, Jiang Z, Xing B (2020). Combined effects of biochar properties and soil conditions on plant growth: a meta-analysis. Sci Tot Environ.

[CR17] Daosukho S, Kongkeaw A, Oengeaw U (2012). The development of durian shell biochar as a nutrition enrichment medium for agricultural purpose: Part 1, chemical and physical characterization. Bull Appl Sci (thailand).

[CR18] das Graças Souza K, de Oliveira MA, Alcantara GU, Paulino GM, de Lima RP, Ferreira OE, Machado AR (2023). Effect of pyrolysis temperature on the properties of the coffee grounds biochar and composition of its leachates. Chem Papers.

[CR22] Fernández-Aparicio M, Cimmino A, Evidente A, Rubiales D (2013). Inhibition of *Orobanche crenata* seed germination and radicle growth by allelochemicals identified in cereals. J Agric Food Chem.

[CR23] Flesch F, Berger P, Robles-Vargas D, Santos-Medrano GE, Rico-Martínez R (2019). Characterization and determination of the toxicological risk of biochar using invertebrate toxicity tests in the state of Aguascalientes. México Appl Sci.

[CR25] Gale NV, Thomas SC (2019). Dose-dependence of growth and ecophysiological responses of plants to biochar. Sci Tot Environ.

[CR26] Gale NV, Sackett TE, Thomas SC (2016). Thermal treatment and leaching biochar alleviates plant growth inhibition from mobile organic compounds. PeerJ.

[CR27] Gale NV, Halim MA, Horsburgh M, Thomas SC (2017). Comparative responses of early-successional plants to charcoal soil amendments. Ecosphere.

[CR28] Gezahegn S, Sain M, Thomas SC (2019). Variation in feedstock wood chemistry strongly influences biochar liming potential. Soil Syst.

[CR29] Gezahegn S, Sain M, Thomas SC (2021). Phytotoxic condensed organic compounds are common in fast but not slow pyrolysis biochars. Biores Technol Rep.

[CR30] Godlewska P, Ok YS, Oleszczuk P (2021). The dark side of black gold: ecotoxicological aspects of biochar and biochar-amended soils. J Hazard Mater.

[CR31] Hale SE, Lehmann J, Rutherford D, Zimmerman AR, Bachmann RT, Shitumbanuma V, O’Toole A, Sundqvist KL, Arp HPH, Cornelissen G (2012). Quantifying the total and bioavailable polycyclic aromatic hydrocarbons and dioxins in biochars. Environ Sci Technol.

[CR33] Himanen M, Prochazka P, Hänninen K, Oikari A (2012). Phytotoxicity of low-weight carboxylic acids. Chemosphere.

[CR35] International Biochar Initiative (IBI) (2015) Standardized product definition and product testing guidelines for biochar that is used in soil (IBI Biochar Standards) version 2.1. https://biochar-international.org/characterizationstandard/. Accessed 11 Nov 2023

[CR36] Jaki T, Hothorn LA (2013). Statistical evaluation of toxicological assays: Dunnett or Williams test—take both. Arch Toxicol.

[CR37] Jeffery S, Abalos D, Prodana M, Bastos AC, Van Groenigen JW, Hungate BA, Verheijen F (2017). Biochar boosts tropical but not temperate crop yields. Environ Res Let.

[CR38] Joseph S, Cowie AL, Van Zwieten L, Bolan N, Budai A, Buss W, Cayuela ML, Graber ER, Ippolito JA, Kuzyakov Y, Luo Y, Ok YS, Palansooriya KN, Shepherd J, Stephens S, Weng Z, Lehmann J (2021). How biochar works, and when it doesn't: a review of mechanisms controlling soil and plant responses to biochar. GCB Bioenergy.

[CR39] Kadota M, Niimi Y (2004). Effects of charcoal with pyroligneous acid and barnyard manure on bedding plants. Sci Hort.

[CR40] Kaur H, Kaushik S (2005). Cellular evidence of allelopathic interference of benzoic acid to mustard (*Brassica juncea* L.) seedling growth. Plant Physiol Biochem.

[CR42] Kloss S, Zehetner F, Dellantonio A, Hamid R, Ottner F, Liedtke V, Schwannigner M, Gerzabek MH, Soja G (2012). Characterization of slow pyrolysis biochars: effects of feedstocks and pyrolysis temperature on biochar properties. J Environ Qual.

[CR43] Kochanek J, Long RL, Lisle AT, Flematti GR (2016). Karrikins identified in biochars indicate post-fire chemical cues can influence community diversity and plant development. PloSOne.

[CR44] Kołtowski M, Oleszczuk P (2015). Toxicity of biochars after polycyclic aromatic hydrocarbons removal by thermal treatment. Ecol Eng.

[CR45] Lehmann J, Gaunt J, Rondon M (2006). Biochar sequestration in terrestrial ecosystems: a review. Mitig Adapt Strateg Glob Change.

[CR46] Lehmann J, Rillig MC, Thies J, Masiello CA, Hockaday WC, Crowley D (2011). Biochar effects on soil biota–a review. Soil Biol Biochem.

[CR47] Liebman M, Sundberg DN (2006). Seed mass affects the susceptibility of weed and crop species to phytotoxins extracted from red clover shoots. Weed Sci.

[CR49] Liu X, Zhang A, Ji C, Joseph S, Bian R, Li L, Pan G (2013). Biochar’s effect on crop productivity and the dependence on experimental conditions—a meta-analysis of literature data. Plant Soil.

[CR50] Lynch JM (1977). Phytotoxicity of acetic acid produced in the anaerobic decomposition of wheat straw. J Appl Bacteriol.

[CR51] Lynch JM (1978). Production and phytotoxicity of acetic acid in anaerobic soils containing plant residues. Soil Biol Biochem.

[CR52] Lynch JM (1980). Effects of organic acids on the germination of seeds and growth of seedlings. Plant Cell Environ.

[CR53] Macías FA, Castellano D, Molinillo JM (2000). Search for a standard phytotoxic bioassay for allelochemicals: selection of standard target species. J Agric Food Chem.

[CR54] Marambe B, Nagaoka T, Ando T (1993). Identification and biological activity of germination-inhibiting long-chain fatty acids in animal-waste composts. Plant Cell Physiol.

[CR56] Mu J, Uehara T, Furuno T (2003). Effect of bamboo vinegar on regulation of germination and radicle growth of seed plants. J Wood Sci.

[CR57] Mu J, Uehara T, Furuno T (2004). Effect of bamboo vinegar on regulation of germination and radicle growth of seed plants II: composition of moso bamboo vinegar at different collection temperature and its effects. J Wood Sci.

[CR59] Oleszczuk P, Jośko I, Kuśmierz M (2013). Biochar properties regarding to contaminants content and ecotoxicological assessment. J Hazard Mater.

[CR60] Olker JH, Elonen CM, Pilli A, Anderson A, Kinziger B, Erickson S, Skopinski M, Pomplun A, LaLone CA, Russom CL, Hoff D (2022). The ECOTOXicology knowledgebase: a curated database of ecologically relevant toxicity tests to support environmental research and risk assessment. Environ Toxicol Chem.

[CR61] Pluchon N, Gundale MJ, Nilsson MC, Kardol P, Wardle DA (2014). Stimulation of boreal tree seedling growth by wood-derived charcoal: effects of charcoal properties, seedling species and soil fertility. Func Ecol.

[CR62] Prill A, Barton LV, Solt ML (1949). Effects of some organic acids on the growth of wheat roots in solutions. Contrib Boyce Thompson Inst.

[CR63] Prochazka P (2008) Acute and subchronic phytotoxicity of volatile fatty acids (VFAs). MSc thesis, University of Jyväskylä, Finland.

[CR64] R Core Team (2023). R: A language and environment for statistical computing. R Foundation for Statistical Computing, Vienna, Austria.

[CR65] Rajkovich S, Enders A, Hanley K, Hyland C, Zimmerman AR, Lehmann J (2012). Corn growth and nitrogen nutrition after additions of biochars with varying properties to a temperate soil. Biol Fert Soils.

[CR66] Rao DN, Mikkelsen DS (1977). Effects of acetic, propionic, and butyric acids on rice seedling growth and nutrition. Plant Soil.

[CR67] Reynolds T (1977). Comparative effects of aliphatic compounds on inhibition of lettuce fruit germination. Ann Bot.

[CR68] Reynolds T (1978). Comparative effects of aromatic compounds on inhibition of lettuce fruit germination. Ann Bot.

[CR69] Ritz C (2010). Toward a unified approach to dose-response modeling in ecotoxicology. Environ Toxicol Chem.

[CR70] Ritz C, Streibig JC (2005). Bioassay analysis using R. J Stat Software.

[CR71] Rogovska N, Laird D, Cruse RM, Trabue S, Heaton E (2012). Germination tests for assessing biochar quality. J Environ Qual.

[CR72] Rombolà AG, Marisi G, Torri C, Fabbri D, Buscaroli A, Ghidotti M, Hornung A (2015). Relationships between chemical characteristics and phytotoxicity of biochar from poultry litter pyrolysis. J Agric Food Chem.

[CR73] Sackett TE, Basiliko N, Noyce GL, Winsborough C, Schurman J, Ikeda C, Thomas SC (2015). Soil and greenhouse gas responses to biochar additions in a temperate hardwood forest. GCB Bioenergy.

[CR74] Sarauer JL, Coleman MD (2018). Biochar as a growing media component for containerized production of Douglas-fir. Can J for Res.

[CR75] Shen X, Meng H, Shen Y, Ding J, Zhou H, Cong H, Li L (2022). A comprehensive assessment on bioavailability, leaching characteristics and potential risk of polycyclic aromatic hydrocarbons in biochars produced by a continuous pyrolysis system. Chemosphere.

[CR77] Somtrakoon K, Chouychai W (2013). Phytotoxicity of single and combined polycyclic aromatic hydrocarbons toward economic crops. Russian J Pl Physiol.

[CR78] Spokas KA, Baker JM, Reicosky DC (2010). Ethylene: potential key for biochar amendment impacts. Plant Soil.

[CR79] Spokas KA, Novak JM, Stewart CE, Cantrell KB, Uchimiya M, DuSaire MG, Ro KS (2011). Qualitative analysis of VOCs on biochar. Chemosphere.

[CR80] Spokas KA, Cantrell KB, Novak JM, Archer DW, Ippolito JA, Collins HP, Boateng AA, Lima IM, Lamb MC, McAloon AJ, Lentz RD, Nichols KA (2012). Biochar: a synthesis of its agronomic impact beyond carbon sequestration. J Environ Qual.

[CR81] Thomas SC (2021). Post-processing of biochars to enhance plant growth responses: a review and meta-analysis. Biochar.

[CR82] Thomas SC, Gale N (2015). Biochar and forest restoration: a review and meta-analysis of tree growth responses. New For.

[CR83] Thomas SC, Halim MA, Gale NV, Sujeeun L (2019). Biochar enhancement of facilitation effects in agroforestry: early growth and physiological responses in a maize-leucaena model system. Agrofor Syst.

[CR84] Tiilikkala K, Fagernäs L, Tiilikkala J (2010). History and use of wood pyrolysis liquids as biocide and plant protection product. Open Agric J.

[CR85] Uddin SMM, Murayama S, Ishmine Y, Tsuzuki E (1995). Studies on sugarcane cultivation. Effects of the mixture of charcoal with pyroligneous acid on cane and sugar yield of spring and ratoon crops of sugarcane (*Saccharum officinarum* L.). Jpn J Trop Agric..

[CR86] Ulbright CE, Pickard BG, Varner JE (1982). Effects of short chain fatty acids on radicle emergence and root growth in lettuce. Plant Cell Environ.

[CR87] Ulbright CE, Pickard BG, Varner JE (1982). Effects of short chain fatty acids on seedlings. Plant Cell Environ.

[CR88] Van Zwieten L, Kimber S, Morris S, Chan KY, Downie A, Rust J, Cowie A (2010). Effects of biochar from slow pyrolysis of papermill waste on agronomic performance and soil fertility. Plant Soil.

[CR90] Viechtbauer W (2010). Conducting meta-analyses in R with the metafor package. J Stat Software.

[CR91] Wang W (1991). Literature review on higher plants for toxicity testing. Water Air Soil Pollut.

[CR92] Wang B, Lehmann J, Hanley K, Hestrin R, Enders A (2015). Adsorption and desorption of ammonium by maple wood biochar as a function of oxidation and pH. Chemosphere.

[CR93] Wilson K (2014) Justus von Liebig and the birth of modern biochar. Biochar J www.biochar-journalorg/en/ct/5

[CR95] Ye L, Camps-Arbestain M, Shen Q, Lehmann J, Singh B, Sabir M (2020). Biochar effects on crop yields with and without fertilizer: a meta-analysis of field studies using separate controls. Soil Use Manage.

[CR96] Yu JQ, Matsui Y (1994). Phytotoxic substances in root exudates of cucumber (*Cucumis sativus* L). J Chem Ecol.

[CR97] Yuan J, Meng J, Liang XEY, Yang X, Chen W (2017). Organic molecules from biochar leacheates have a positive effect on rice seedling cold tolerance. Front Plant Sci.

