# Supplementary material for: Phytotoxicity and hormesis in common mobile organic compounds in leachates of wood-derived biochars
Source: Biochar. 2024 May 22;6(1):51. doi: 10.1007/s42773-024-00339-w (PMC11111554; doi:10.1007/s42773-024-00339-w)
Supplement: Supplementary file 4 — Supplementary Material 4. [file 42773_2024_339_MOESM4_ESM.docx]

Supplemental Table 1. List of concentrations tested for organic compounds used in phytotoxicity assays. Ranges of concentrations were adjusted to reflect differences in solubility.

| Compound | Concentrations tested (mmol L^-1^) |
| --- | --- |
| 2,4-Di-tert-butylphenol | 0.00001, 0.00003, 0.0001, 0.0003, 0.001, 0.003, 0.01, 0.03, 0.1 |
| 2-ethylbutyric acid | 0.01, 0.03, 0.1, 0.3, 1, 3, 10, 30, 100 |
| Acetic acid | 0.01, 0.03, 0.1, 0.3, 1, 3, 10, 30, 100 |
| Acetone | 0.01, 0.03, 0.1, 0.3, 1, 3, 10, 30, 100, 300, 1000, 3000, 10000 |
| Benzene | 0.01, 0.03, 0.1, 0.3, 1, 3, 10 |
| Butyric acid | 0.01, 0.03, 0.1, 0.3, 1, 3, 10, 30, 100 |
| Caproic acid | 0.01, 0.03, 0.1, 0.3, 1, 3, 10, 30, 100 |
| Methyl ethyl ketone | 0.01, 0.03, 0.1, 0.3, 1, 3, 10, 30, 100 |
| Methyl salicylate | 0.01, 0.03, 0.1, 0.3, 1, 3 |
| Phenol | 0.01, 0.03, 0.1, 0.3, 1, 3, 10, 30, 100 |
| Propionic acid | 0.01, 0.03, 0.1, 0.3, 1, 3, 10, 30, 100 |
| Toluene | 0.01, 0.03, 0.1, 0.3, 1, 3 |
| Valeric acid | 0.01, 0.03, 0.1, 0.3, 1, 3, 10, 30, 100 |

Supplemental Table 2. List of organic compounds (IUPAC names followed by common names where applicable) identified in qualitative GC-MS analyses of leachates of 13 tested biochars, arranged in decreasing order by frequency of occurrence. Compounds detected in aqueous biochar leachates are marked “*”.

| **Compound** | **Frequency** |
| --- | --- |
| *Acetic acid | 13/13 |
| *Pentanoic acid (valeric acid) | 13/13 |
| *2,4-Di-tert-butylphenol | 13/13 |
| *Methyl salicylate (oil of wintergreen) | 12/13 |
| *Hexanoic acid (caproic acid) | 12/13 |
| *2-Ethyl butanoic acid (2-ethylbutyric acid; acetic acid, diethyl-) | 11/13 |
| Benzaldehyde, 4-hydroxy-3,5-dimethoxy- | 10/13 |
| Diethyl phthalate | 6/13 |
| *p-Pentylacetophenone | 6/13 |
| Benzofuran | 6/13 |
| 4-Methoxycinnamaldehyde | 6/13 |
| *Propanoic acid (propionic acid) | 5/13 |
| Fluorene | 5/13 |
| *1,3,3-Trimethyl-2-oxabicyclo[2.2.2]octane (eucalyptol) | 5/13 |
| *1,3-Butadiyne | 5/13 |
| Isopropylmyristate | 5/13 |
| 2,3,6-Trichlorobenzaldehyde | 5/13 |
| Phenol, 2-methoxy-4-propyl- | 5/13 |
| *Benzoic acid | 4/13 |
| Butanoic acid (butyric acid) | 4/13 |
| Benzaldehyde | 4/13 |
| *Benzophenone | 4/13 |
| Nonanoic acid (pelargonic acid) | 4/13 |
| Isopropylpalmitate | 4/13 |
| Furan, 2,5-dimethyl- | 4/13 |
| Benzoic acid, 4-formyl- | 4/13 |
| *Butanamide, N-(2-methoxyphenyl)-3-oxo- | 4/13 |
| *2,5-Cyclohexadiene-1,4-dione, 2,6-bis(1,1-dimethylethyl)- | 4/13 |
| Apocynin | 3/13 |
| Benzothiazole | 3/13 |
| *Diphenylether | 3/13 |
| *Acetamide, 2-fluoro- | 3/13 |
| 1,2-Benzenediol, 3-methyl- | 3/13 |
| Benzene, 1-methoxy-3-phenoxy- | 3/13 |
| Hexadecanoicacid, methylester | 3/13 |
| *Phenol, 2-methyl-6-(2-propenyl)- | 3/13 |
| Nonanal | 2/13 |
| *Flamenol | 2/13 |
| Pyridine | 2/13 |
| *Warfarin | 2/13 |
| *1-Butanol | 2/13 |
| Benzoic acid | 2/13 |
| Butanoic acid (butyric acid) | 2/13 |
| Propanoic acid (propionic acid) | 2/13 |
| *2(1H)-Pyridinone | 2/13 |
| Phenol, 2-methoxy- | 2/13 |
| *7-Amino-3-phenylcoumarin | 2/13 |
| *Naphthalene, 1-isocyano- | 2/13 |
| Bis(2-ethylhexyl)phthalate | 2/13 |
| Hexadecanoicacid, butylester | 2/13 |
| Heptane, 2,2,4,6,6-pentamethyl- | 2/13 |
| Acridine, 9,10-dihydro-9,9-dimethyl- | 2/13 |
| Benzene, 1-methoxy-4-(2-phenylethenyl)- | 2/13 |
| *7H-Dibenzo(a,g)carbazole, 12,13-dihydro- | 2/13 |
| Benzenamine, N,N-dimethyl-4-[(3-methylphenyl)azo]- | 2/13 |
| 2,5-Cyclohexadiene-1, 4-dione,2,6-bis(1,1-dimethylethyl)- | 2/13 |
| *Cyclopenta[g]-2-benzopyran, 1,3,4,6,7,8-hexahydro-4,6,6,7,8,8-hexamethyl- | 2/13 |
| 2-Hexene | 1/13 |
| Furfural | 1/13 |
| Lapachol | 1/13 |
| Allantoin | 1/13 |
| Harmaline | 1/13 |
| 2-Decanone | 1/13 |
| 1H-Indazole | 1/13 |
| *3-Pentanone | 1/13 |
| Caprolactam | 1/13 |
| Metharbital | 1/13 |
| 1-Butanamine | 1/13 |
| Dibenzofuran | 1/13 |
| Fumaric acid | 1/13 |
| Tropacocaine | 1/13 |
| 2-Propenamide | 1/13 |
| Octanoic acid | 1/13 |
| Benzeneselenol | 1/13 |
| Pentanoic acid (valeric acid) | 1/13 |
| Sulfacarbamide | 1/13 |
| Hexylresorcinol | 1/13 |
| Methylstearate | 1/13 |
| Dimethylsulfone | 1/13 |
| *o-Phenanthroline | 1/13 |
| 1,1-Dicyanoethane | 1/13 |
| *o-Hydroxybiphenyl | 1/13 |
| Hydrazine,phenyl- | 1/13 |
| *n-Hexylmethylamine | 1/13 |
| Succinic anhydride | 1/13 |
| Naphtho[2,1-b]furan | 1/13 |
| Tetraethylsilicate | 1/13 |
| Thiophene, 3-bromo- | 1/13 |
| 2,4-Dimethoxytoluene | 1/13 |
| 6-Bromohexanenitrile | 1/13 |
| Cyclohexane, pentyl- | 1/13 |
| Methane, tetranitro- | 1/13 |
| 4-Acetoxyacetophenone | 1/13 |
| *9H-Fluorene, 9-bromo- | 1/13 |
| *Mercury, bromomethyl- | 1/13 |
| 4-Fluorodiphenylether | 1/13 |
| Acetaldehyde, hydroxy- | 1/13 |
| Benzoic acid, 2-nitro- | 1/13 |
| N-Nitrosodiphenylamine | 1/13 |
| Pentadecane, 2-methyl- | 1/13 |
| 1H-Imidazole, 1-methyl- | 1/13 |
| *4-Methoxycinnamaldehyde | 1/13 |
| Benzaldehyde, 4-ethoxy- | 1/13 |
| *Benzoic acid, 2-methyl- | 1/13 |
| *Benzoic acid, 3-methyl- | 1/13 |
| Benzoic acid, 4-formyl- | 1/13 |
| *Hexamethylphosphoramide | 1/13 |
| Hydrazinecarbothioamide | 1/13 |
| Methane, bromotrinitro- | 1/13 |
| 2-Fluorenecarboxaldehyde | 1/13 |
| 3-Methylsalicylhydrazide | 1/13 |
| 9H-Carbazole, 9-nitroso- | 1/13 |
| Benzene, (fluoromethyl)- | 1/13 |
| Butanoic acid, 2-methyl- | 1/13 |
| 1,2-Ethanediol,diacetate | 1/13 |
| 4-Methyl-2-pentylacetate | 1/13 |
| Propanoic acid, 2-methyl- | 1/13 |
| 2,5-Dihydroxypropiophenone | 1/13 |
| 3,5-Diamino-1,2,4-triazole | 1/13 |
| 9-(Chloromethyl)anthracene | 1/13 |
| Dibutylmethanephosphonate | 1/13 |
| Phenol, 2-methoxy-4-propyl- | 1/13 |
| 2,4,6-Trimethoxyacetophenone | 1/13 |
| 2H-Inden-2-one, 1,3-dihydro- | 1/13 |
| Benzene, pentafluoromethoxy- | 1/13 |
| 1H-Pyrazole, 1,3,5-trimethyl- | 1/13 |
| Methacrylic acid, ethylester | 1/13 |
| (R)-(+)-3-Methylcyclopentanone | 1/13 |
| Benzene, 1-methyl-2,3-dinitro- | 1/13 |
| Cyclohexane, (2-methylpropyl)- | 1/13 |
| *Diisopropyl methanephosphonate | 1/13 |
| Heptane, 2,2,4,6,6-pentamethyl- | 1/13 |
| 2-Cyclohexen-1-one, 4,4-dimethyl- | 1/13 |
| 2-Propenamide, N-(hydroxymethyl)- | 1/13 |
| 2,5-Furandione, dihydro-3-methyl- | 1/13 |
| Benzaldehyde, 3-ethoxy-2-hydroxy- | 1/13 |
| Benzene, 1-(bromomethyl)-2-chloro- | 1/13 |
| *4-Piperidinone, 2,2,6,6-tetramethyl- | 1/13 |
| Phenol, 4,4'-(1-methylethylidene)bis- | 1/13 |
| Methane, (methylsulfinyl)(methylthio)- | 1/13 |
| 1-Naphthalenecarboxaldehyde, 2-hydroxy- | 1/13 |
| Benzoic acid, 4-hydroxy-, n-heptylester | 1/13 |
| *Phosphoramidic acid, phenyl-, diphenylester | 1/13 |
| 1,2-Benzenedicarboxylic acid, monobutylester | 1/13 |
| 1(2H)-Naphthalenone, 3,4-dihydro-5,7-dimethyl- | 1/13 |
| *Oxirane, [[4-(1,1-dimethylethyl)phenoxy]methyl]- | 1/13 |
| Acetic acid, (2,4-dichlorophenoxy)-, 2-methylpropylester | 1/13 |
| Phosphorodiamidicacid, tetramethyl-, pentachlorophenylester | 1/13 |
| Cyclohexanecarboxylicacid, 4-butyl-, 4-ethoxyphenylester, trans- | 1/13 |
